# Gene Expression Profiling of PDGFRA Mutant GIST Reveals Immune Signatures as a Specific Fingerprint of D842V Exon 18 Mutation

**DOI:** 10.3389/fimmu.2020.00851

**Published:** 2020-06-02

**Authors:** Valentina Indio, Gloria Ravegnini, Annalisa Astolfi, Milena Urbini, Maristella Saponara, Antonio De Leo, Elisa Gruppioni, Giuseppe Tarantino, Sabrina Angelini, Andrea Pession, Maria Abbondanza Pantaleo, Margherita Nannini

**Affiliations:** ^1^“Giorgio Prodi” Cancer Research Center (CIRC), University of Bologna, Bologna, Italy; ^2^Department of Pharmacy and Biotechnology, University of Bologna, Bologna, Italy; ^3^Department of Morphology, Surgery and Experimental Medicine, University of Ferrara, Ferrara, Italy; ^4^Biosciences Laboratory, Istituto Scientifico Romagnolo per lo Studio e la Cura dei Tumori (IRST) IRCCS, Meldola, Italy; ^5^Department of Experimental, Diagnostic and Specialty Medicine, University of Bologna, Bologna, Italy; ^6^Pathology Unit, Department of Experimental, Diagnostic and Specialty Medicine, S.Orsola-Malpighi Hospital, University of Bologna, Bologna, Italy; ^7^Medical Oncology Unit, S.Orsola-Malpighi University Hospital, Bologna, Italy

**Keywords:** gastrointestinal stromal tumor, GIST, PDGFRA, D842V, tumor infiltrating lymphocytes, IFN-γ signaling pathway, immunotherapy, checkpoint inhibitor

## Abstract

Platelet Derived Growth Factor Receptor Alpha (PDGFRA) mutations occur in only about 5–7% of gastrointestinal stromal tumors (GIST), notably with alterations on exons 12/14/18. The most frequent PDGFRA mutation is the exon 18 D842V, which is correlated to specific clinico-pathological features, such as primary imatinib resistance and higher indolence. Here, we present a gene expression profile (GEP) comparison of D842V vs. PDGFRA with mutations other than D842V (non-D842V). GEP was followed by *in silico* bioinformatic analysis aimed at evaluating differential expression, tumor microenvironment composition and pathway enrichment. We found a large set of oncogenes, transcription factors and nuclear receptors downregulated in the D842V mutant. Conversely, D842V showed a significant enrichment of immune- and interferon- related gene signatures. Differences in tumor microenvironment composition were also highlighted, including a higher abundance of CD8+ T-cells and an overexpression of the T cell-inflamed signature in the D842V mutant subgroup, which is predictive of immunotherapy response. PDGFRA D842V vs. non-D842V GIST display a different expression profile, with a prominent immunological signature, that could represent a proof of principle for testing immunotherapeutic strategies in this drug-orphan subset of GIST.

## Introduction

Platelet Derived Growth Factor Receptor Alpha (PDGFRA) mutations occur in only about 5–7% of gastrointestinal stromal tumors (GIST), and mainly involve the A-loop encoded by exon 18 (~5%), or more rarely the JM domain encoded by exon 12 (~1%), or the ATP binding domain encoded by exon 14 (<1%) (1, 2). In particular, the substitution at position 842 in the A-loop of an aspartic acid (D) with a valine (V), recognized as D842V, is the most frequent mutation and the one widely known to confer primary resistance to imatinib by changing the kinase domain conformation, which negatively affects imatinib binding (2–8). Thus, patients with D842V mutant GIST have a very low rate of clinical benefit from imatinib treatment (5–8). Moreover, the D842V mutant kinase is also strongly resistant to sunitinib *in vitro* and limited clinical data suggests that sunitinib has low activity against D842V dependent GIST (9).

Therefore, those patients do not benefit from standard TKI therapy and currently represent one of the main unmet medical needs in GIST management.

Crenolanib, a known a potent inhibitor of PDGFRA and PDGFRB, and avapritinib, a highly selective and potent KIT/PDGFRA inhibitor, have shown promising anti-proliferative activity against D842V mutant GIST (10, 11). However, no actionable recurrent molecular events of clinical significance in D842V mutant GIST have been found, so the potential therapeutic scenario of this rare subset of GIST remains still limited (12).

Recently, it has been shown that GIST show a gene expression profile suggestive of possible response to immune checkpoint inhibitors (13) and, in particular, that PDGFRA mutant GIST displays a more prominent immune cell pathway when compared to KIT mutant GIST (14). In particular, it has been found that PDGFRA mutant GIST displays more immune cells with increased cytolytic activity; express higher levels of many chemokines, such as CXCL14; exhibit more diverse driver-derived neoepitope-HLA binding proteins; and have additional immune features of high PD-1 and PD-L1 expressing tumors. Those findings could pave the way for a rational basis for exploring an immune-treatment approach in this molecular subset of GIST.

In this intriguing scenario, the aim of this study was to specifically evaluate the immune-profile of D842V mutant GIST compared to non-D842V mutant GIST, in order to better understand if the prominent immune features belong to all PDGFRA mutant GIST or if it is a specific peculiar fingerprint of D842V mutants, widely recognized as the drug-orphan subset of GIST.

## Methods

### Patients and Tumor Samples

Fresh surgical specimens of 10 patients with untreated, primary gastric GIST were collected immediately upon resection and frozen in liquid nitrogen. The clinical and pathological characteristics are summarized in Table 1. GIST diagnosis was based on histologic evaluation and on immunohistochemistry of CD117 and DOG1 as reviewed by expert pathologists. All patients harbored a gain of function mutation in the PDGFRA gene. Specifically, 5 patients had a D842V exon 18 PDGFRA mutation and 5 had non-D842V PDGFRA mutations (in particular, 3 had alterations on exon 12, 1 on exon 14, and 1 on exon 18 non-D842V).

**Table 1:** Patient's characteristics.

**Patient ID**	**Age (range)**	**Gender**	**Site**	**Size (cm)**	**Mitotic count (HPF)**	**Risk classification**	**Disease status**	**Last follow up**	**Tumor tissue type**	**Molecular analysis**
GIST140	41–45	F	Stomach	15	3/50	High	Localized	AWOD	Fresh	D842V
GIST165	51–55	M	Stomach	12	2/50	Intermediate	Localized	AWOD	Fresh	D842V
GIST138	71–75	F	Stomach	7	8/50	High	Localized	AWOD	Fresh	D842V
GIST142	66–70	M	Stomach	3	5/50	Very low	Localized	AWOD	Fresh	D842V
GIST136	76–80	M	Stomach	4.5	6/50	Intermediate	Localized	DNFD	Fresh	D842V
GIST12	66–70	F	Stomach	NA	NA	NA	Localized	NA	Fresh	Exon 18 K646E
GIST168	56–60	F	Stomach	5.5	4/50	Intermediate	Localized	AWOD	Fresh	Exon 12 c.1698_1712del15 (p.S566_E571>R)
GIST05	66–70	F	Stomach	7	4/50	Low	Localized	AWOD	Fresh	Exon 12 del 16117-20 CCCG + ins 16124 TC + del 16124-30 GGACATG
GIST15	61–65	NA	Stomach	NA	NA	NA	Localized	NA	Fresh	Exon 18 del DIMH842-845
GIST26	46–50	NA	Stomach	NA	NA	NA	Localized	NA	Fresh	Exon 12 V561D

*AWOD, alive without disease*.

*DNFD, dead not for disease*.

### Gene Expression Profile

Whole transcriptome expression profile was evaluated using a GeneChipTM WT PLUS Reagent Kit (Applied Biosystems) performed on a NextSeq500 Illumina platform (Illumina, Inc., San Diego, CA, USA). Total RNA was extracted from fresh frozen tumor specimens using an RNeasy Mini Kit (Qiagen, Milan, Italy). The quality and quantity of RNA were determined with a UV–Vis spectrophotometer at 260 nm/280 nm absorbance. Integrity of RNA was checked using an RNA6000 Pico Kit (Agilent) and all samples had RIN>7. Whole transcriptome expression profile was determined using a microarray Clariom S chip (Affymetrix, ThermoFisher), following the manufacturer's instructions. Briefly, 100 ng of total RNA was used to generate cDNA, then fragmented and labeled cDNA was hybridized to a Human Clariom S array for 16 h at 45°C. Arrays were washed, stained and then scanned using the Affymetrix Gene Chip Scanner 7G and CEL Intensity files were generated by Affymetrix GeneChip Command Console Software (AGCC, Thermo Fisher).

### Bioinformatic Analysis

Gene expression profiling analysis was implemented with R-bioconductor packages (https://www.bioconductor.org/). CEL files were analyzed by adopting the Robust Multi-Array Average algorithm (*rma* function, *oligo* package) that was applied to background-subtraction, normalization and log-transformation of signals intensity.

Genes with a log-transformed signal lower that 5 in more than 7/10 samples were filtered, as well as genes with IQR < 0.3. The evaluation of differential expressed genes between D824V vs. non-D842V mutant GIST was performed by fitting a linear model, followed by an empirical Bayes moderate unpaired t-statistic (*lmFit* and *eBayes* functions, *limma* package). Principal component analysis was performed with the *prcomp* function of the *stats* package and the corresponding projections of the 1st, 2nd, and 3rd components were plotted with the function *plot3d* of *rgl* package. Gene expression profiles were adopted to perform the Gene Ontology (GO) enrichment analysis with the WEB-based GEne SeT AnaLysis Toolkit (WebGestalt) web application (http://www.webgestalt.org/) selecting “Homo sapiens” as the organism, “Gene Set Enrichment Analysis (GSEA)” as the method and “geneontology” and “Biological Process nonRedundant” as the functional database. Moreover, the enrichment of the gene pathway included in the curated Molecular Signatures Database (MSigDB) (http://software.broadinstitute.org/gsea/msigdb/collections.jsp#C2) was evaluated with the Gene Set Enrichment Analysis (GSEA) preranked tool from Broad Institute (http://software.broadinstitute.org/gsea/index.jsp) set to 1,000 permutations and the default parameters. Both analyses were performed on the list of differentially expressed genes that were pre-ranked according to the score *S* = log10(*p*-value)^*^(fold change sign).

The evaluation of tumor microenvironment composition was done using the web tool CIBERSORT (https://cibersort.stanford.edu/) adopting LM22 as the reference, with gene expression signatures consisting of 22 distinct immune cell types. We ran the tool in both absolute and relative mode, with 100 permutations and disabling the quantile normalization.

All of the heatmaps were built with the R-bioconductor package *pheatmap*, adopting the “euclidean” metric of distance and the clustering method “ward.D.”

### Validation of Gene Expression

Four of the most deregulated genes, BCL6, FOXO1, NRAS and NR4A3, were validated through qRT-PCR. cDNA was obtained using a High-Capacity RNA-to-cDNA Kit (Applied Biosystem) and the expression level was evaluated through the 7900HT Fast Real-Time PCR System (Applied Biosystems). Fold change was evaluated using the DDCt method, using GAPDH and HBMS as housekeeping genes. The primers used were: BCL6_Fwd 5′ - CTCCGGAGTCGAGACATCTT – 3′; BCL6_Rev 5′ - GCTATAGAACAGGCCACTGC – 3′; FOXO1_Fw 5′- TCACGCTGTCGCAGATCTAC−3′; FOXO1_Rev 5′ – TTGAATTCTTCCAGCCCGCC – 3′; NRAS_Fw, 5′- ACAGTGCCATGAGAGACCAA – 3′; NRAS_Rev 5′ TCGCTTAATCTGCTCCCTGT−3′; NR4A3 _Fwd 5′ - GACGTCGAAACCGATGTCAG – 3′; NR4A3 Rev 5′- GGGCTCTTTGGTTTGGAAGG – 3′; GAPDH_Fw 5′-CGGGAAGCTTGTCATCAAT-3′ and GAPDH_Rev 5′- GACTCCACGACGTACTCAGC-3′, HBMS Fw-5′ TGTGGTGGGAACAGCTC-3′ and HBMS_rev 5′-TGTTGAGGTTTCCCCGAAT-3′.

## Results

Gene expression profile (GEP) was assessed by performing microarray analysis experiments in a series of 10 PDGFRA mutant GIST patients either carrying a genomic alteration on exon 18 D842V (5 out of 10 samples) or non-D842V mutations, including 2 patients harboring point variants (exon 12 V561D, exon 14 K646E) and three patients showing insertions/deletions (indel) either in exon 12 or exon 18. Molecular lesions together with patient's characteristics are listed in Table 1.

The comparison of transcription profiles between the D84V and non-D842V PDGFRA mutant subgroups showed considerably different expression patterns. Adopting the significance threshold of *p* < 0.05 we found 1,153 significantly modulated genes (Supplementary Table S1) of which 968 were differentially expressed with |logFC|>0.5, specifically 312 over-expressed and 656 down-regulated in the D842V samples. The expression divergence was also highlighted by principal component analysis (PCA), performed in an unsupervised manner, by which the separation between D842V and non-D842V is evident in the third component (Supplementary Figure S1). Included in the set of differentially modulated transcripts we found a relatively high number of genes included in the Oncogene Database (http://ongene.bioinfo-minzhao.org/browse_gene.html#protein). In particular, 59 oncogenes emerged as significantly downregulated in the D842V samples with respect to the non-D842V group (Supplementary Table S2). Among them we found the proto-oncogenes ABL1 (*p* = 0.0101; log2FC = −0.54), BRAF (*p* = 0.0204; log2FC = −0.51), NRAS (*p* = 0.0314; log2FC = −0.65), CBL (*p* = 0.0346; log2FC = −0.65), the growth factor CTGF (*p* = 0.0049; log2FC = −1.55) and the transcriptional factors/repressors BCL11A (*p* = 0.0002; log2FC = −2.02), BCL6 (*p* = 0.0076; log2FC = −0.98), ETV3 (*p* = 0.0066; log2FC = −0.71), EWSR1 (*p* = 0.0220; log2FC = −0.52), FOXO1 (*p* = 0.0131; log2FC = −0.62). Moreover, we also found in the D842V mutants a significantly lower level of nuclear receptors (not listed in the Oncogene Database) including NR4A1 (*p* = 0.0104; log2FC = −1.57), NR4A2 (*p* = 0.0016; log2FC = −1.53), NR4A3 (*p* = 0.0008; log2FC = −1.61) and NR3C1 (*p* = 0.0047; log2FC = −0.76). Interestingly, the PDGFRA gene itself also appeared differentially downregulated in the D842V group even though there was a smaller difference between the two groups of patients (*p* = 0.0366; log2FC = −0,47) (Supplementary Figure S2A).

**Figure 1:**
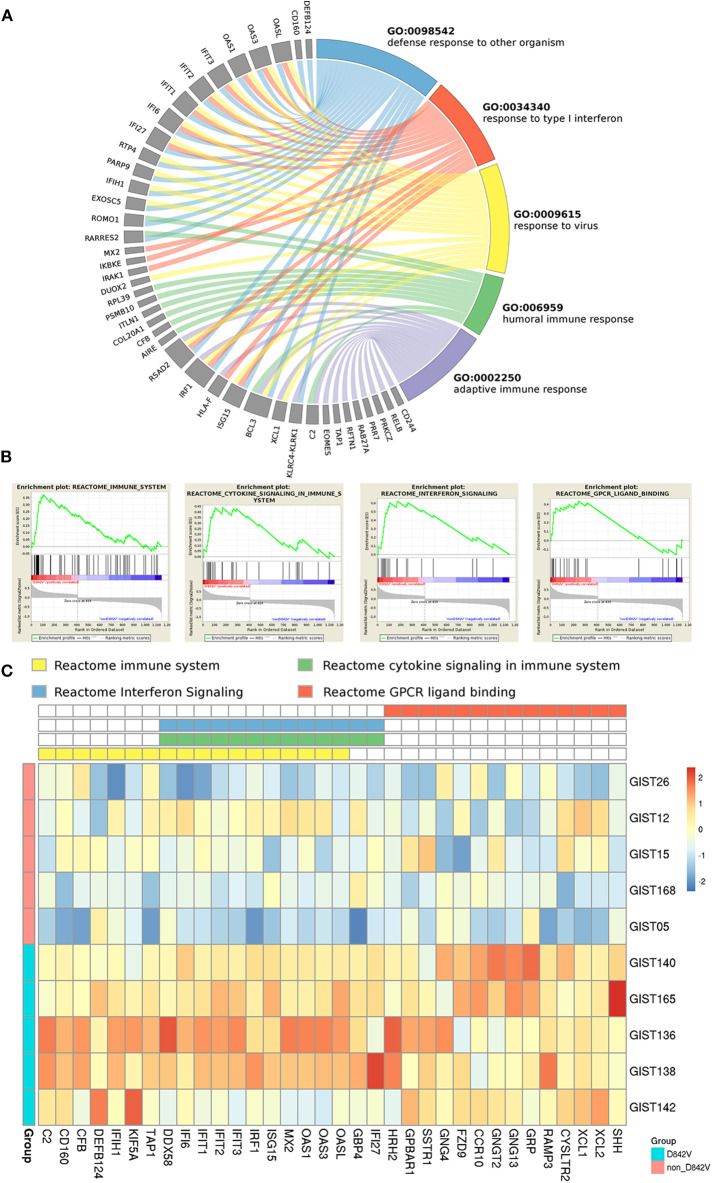
Pathway enrichment of PDGFRA D842V mutant GIST. **(A)** Gene Ontology biological process analysis (performed with WebGestalt) highlighted immune related GO terms significantly enriched (FDR < 0.05) in D842V mutant samples, including “response to type I interferon,” “defense response to other organism,” “response to virus,” “adaptive immune response” and “humoral immune response”. The circos plot shows the correspondence between genes and biological process. **(B)** Consistently, GSEA analysis revealed 4 REACTOME signatures significantly enriched (FDR < 0.05) that are involved in immune modulations. **(C)** The leading edge genes included in these signatures are plotted in the heatmap in which the expression level in both D842V and non-D842V samples is shown.

To assess the robustness of the analyses, we used q-RT-PCR to validate a few genes randomly selected among the most significantly deregulated ones. Specifically, we tested BCL6, FOXO1, NRAS and NR4A3. Supplementary Figure S2B summarizes the results of q-RT-PCR; in agreement with data from GEP, the non-D842V subgroup showed an upregulation of BCL6 (Fold Difference = 0.28), FOXO1 (Fold Difference = 0.95), NRAS (Fold Difference = 0.49), and NR4A3 (Fold Difference = 1.55), with respect to the D842V group. The set of 1153 differentially expressed genes was adopted to perform GO enrichment analysis with the WebGestalt tool, and the GSEA from the Broad Institute was used to evaluate gene pathway enrichment included in the curated MSigDB (Supplementary Tables S3, S4, respectively). Interestingly, looking at the D842V subgroup, both analyses showed very similar results: we found significantly enriched GO-terms linked to the immune system (such as “response to type I interferon,” “defense response to other organism,” “response to virus,” “adaptive immune response,” “interferon-gamma production,” etc.) (Figure 1A) as well as several Reactome signatures related to the immune response including “Interferon signaling,” “Immune system” and “Cytokine signaling in immune system” (Figures 1B,C).

On the other hand, the non-D842V subgroup was enriched in more general and aspecific GO-terms and signatures (Supplementary Figures S3A,B).

**Figure 2:**
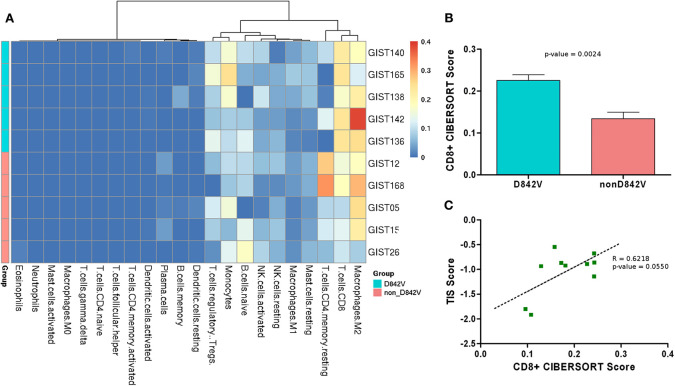
**(A)** Heatmap representing the composition of the tumor microenvironment absolute abundance predicted by CIBERSORT analysis (absolute abundance). D842 and non-D842V mutant GIST are labeled in cyan and pink, respectively. **(B)** Boxplot representing the CD8+ T-cell abundance that appears significantly higher in the D842V compared to the non-D842V mutant GIST. **(C)** Correlation between TIS score and CD8+ T-cell abundance.

The gene expression profiles were also analyzed with CIBERSORT to evaluate the tumor microenvironment composition (Supplementary Table S5). Overall, the analysis showed M2 macrophages, CD8+ T-cells and CD4+ T-cells as the most abundant hematopoietic cell population in the tumor infiltrate; in addition a moderate presence of monocytes and regulatory T-cells (Treg) was predicted (Figure 2A). Interestingly, a significantly higher abundance of CD8+ T-cells was found in the D842V patients compared to the non-D842V ones (Figure 2B). The data also showed some differences in the presence of Tregs (more abundant in the D842V group) and CD4^+^ T-cells (less abundant in the D842V group), that unfortunately did not reach statistical significance, probably due to the small number of samples (Supplementary Figures S4A,B).

Further to the CIBERSORT results, the transcriptome profiles were additionally investigated to study the T cell-inflamed signature (TIS) described by Ayers et al. as characteristic of the expression profile of neoplasms that are sensitive to the PD-1 checkpoint blockade (15).

This 18-gene signature, composed of IFN-γ signaling genes, cytokines, cytotoxic effectors and antigen-presenting genes, was analyzed in order to find the TIS score, a unique value measuring the signature expression level, as previously done by Pantaleo et al. (13) (Supplementary Table S6). Combining the CIBERSORT results with TIS analysis we found that the TIS score positively correlated with the absolute abundance predicted by CIBERSORT (*R* = 0.8640, *p* = 0.0013) (Supplementary Figure S5), and notably also with the CD8+ T-cell abundance (*R* = 0.6218; *p* = 0.0550) (Figure 2C).

## Discussion

In the fast-growing era of immunotherapy, some findings have provided the rationale for implementing immunotherapeutic strategies in the therapeutic scenario of GIST (13, 14, 16–19). Recently, it has been shown that PDGFRA mutant GIST display a more prominent immune cell pathway when compared to KIT mutant GIST, suggesting that immunotherapeutic strategies in GIST could be molecularly-driven (14).

However, it is known that PDGFRA mutant GIST are themselves heterogeneous in clinical behavior and imatinib-sensitiveness, according to the exon involved and to what kind of mutation occurred (2). Therefore, in the present study we profiled, by gene expression analysis, 10 samples of untreated primary gastric PDGFRA mutant GIST, half carrying a D842V mutation and half carrying mutations other than D842V, supposing that the different clinical behavior of these two PDGFRA mutant subgroups could be supported by a different biological background. Indeed, it has been widely recognized that mutant PDGFRA GIST, mostly represented by D842V mutants, correlated with a very favorable disease outcome (20–22). Moreover, even if a conclusion cannot be drawn due to the limited number of cases, it has been found that the D842V mutant GIST, along with those carrying PDGFRA exon 12 and exon 14 mutations, display a more favorable prognosis, while those with exon 18 non-D842V mutations have a more aggressive behavior (22).

Interestingly, we found considerably different expression patterns in D842V mutant GIST compared to non-D842V mutant GIST. In particular, the D842V mutant gene profile presented a lower expression of a large subset of oncogenes and transcription factors, such as PDGFRA, BRAF, BCL6, BCL11A, NRAS, ETV3, NR4A1,NR4A2, NR4A3, and NR3C1. This evidence may support the higher indolence previously observed in the D842V mutant with respect to the other molecular GIST subgroups. Unfortunately, we are not able to assess the differences in terms of aggressiveness nor mitotic activity in our samples due to the lack of complete clinical information. However, this observation could represent a subject to be further investigated in a larger GIST series focusing specifically on the prognostic landscape of gene expression.

Beyond this aspect, the present study highlighted that the D842V mutant exhibits a notable enrichment of immune-signature and an increased TIS score with respect to non-D842V GIST. Consistently, the analysis of tumor microenvironment composition showed a significantly higher abundance of CD8+ T-cells and Tregs, and a lower rate of CD4+ T-cells.

Despite what it looks like, our observations are not in contrast with the study by Vitiello et al. which highlighted not only an unquestionable higher immunogenicity of the PDGFRA mutant, but also indicated the presence (mostly in the D842V mutant) of neoepitopes with a high binding affinity to common HLA types (14). Actually, our study goes further into PDGFRA mutant GIST by exploring the differences between the D842V and non-D842V gene expression profiles and surprisingly shows that GIST with the D842V mutation is the subgroup driving the discoveries previously made, probably because they are, as matter of fact, the most frequent mutation in PDGFRA mutant GIST. From our study we can hypothesize that the high number of high affinity neoepitopes created by the D842V mutation may lead to an increased recruitment of T cells, which in turn induces the IFN-γ signature and PD-L1 expression in the tumor cells.

Taking all of these findings together this is the first study, to the best of our knowledge, showing that within the PDGFRA mutant GIST, the D842V mutant subset displays a distinct gene expression profile, deeply different from the other PDGFRA mutant subsets, that could likely justify their different clinical behaviors. Firstly, the marked immunogenicity of PDGFRA mutant GIST as shown by Vitiello et al. (which by our findings may be only restricted to the D842V mutant), together with the lack of an oncogene-signature, could in part explain the known indolent course of this subset of GIST, irrespective to the recognized prognostic factors.

Secondly, this immunogenicity may represent a proof of principle for testing immunotherapeutic strategies alone or in combination with novel compounds still under evaluation, such as crenolanib and avapritinib, in metastatic D842V mutant GIST, given their proven primary resistance to imatinib and sunitinib, and the lack of effective treatment options at this time.

We know that the main limitation of the study is the small sample size analyzed, due to the rarity of this genomically defined population of GIST. Therefore, as future perspective, our intent will be to confirm the data on a larger sample size, and correlate them with clinical follow up data. As well, a comparison between primary and metastatic tissue will be considered, in order to evaluate the degree of immunogenicity in relation to the disease status.

In conclusion, these preliminary data, even if limited by the small size, confirm the immunological fingerprint of D842V mutant GIST and may represent another brick in the wall of immunotherapy for GIST.

## Data Availability Statement

Gene expression profiling data are available upon request by contacting Dr Valentina Indio at valentina.indio2@unibo.it.

## Ethics Statement

This study was approved by the local institutional ethical committee of Azienda Ospedaliero-Universitaria Policlinico S.Orsola-Malpighi (number 113/2008/U/Tess). The patients/participants provided their written informed consent to participate in this study.

## Author Contributions

MN, VI, GR, and MP conceived and designed the work and drafted the manuscript. VI, GR, AA, GT, MS, and MU supported the data analysis. AD performed the morphological and immunohistochemical analyses. EG performed the molecular analyses. MN, VI, GR, AA, SA, AP, and MP helped with drafting the manuscript and the final revision.

## Conflict of Interest

MP declares research grant support by Novartis for GIST research. The remaining authors declare that the research was conducted in the absence of any commercial or financial relationships that could be construed as a potential conflict of interest.
