# Supplementary material for: Gene Expression Profiling of PDGFRA Mutant GIST Reveals Immune Signatures as a Specific Fingerprint of D842V Exon 18 Mutation
Source: Front Immunol. 2020 Jun 2;11:851. doi: 10.3389/fimmu.2020.00851 (PMC7326057; doi:10.3389/fimmu.2020.00851)
Supplement: Supplementary file 1 [file Presentation_1.pptx]

## Slide 1
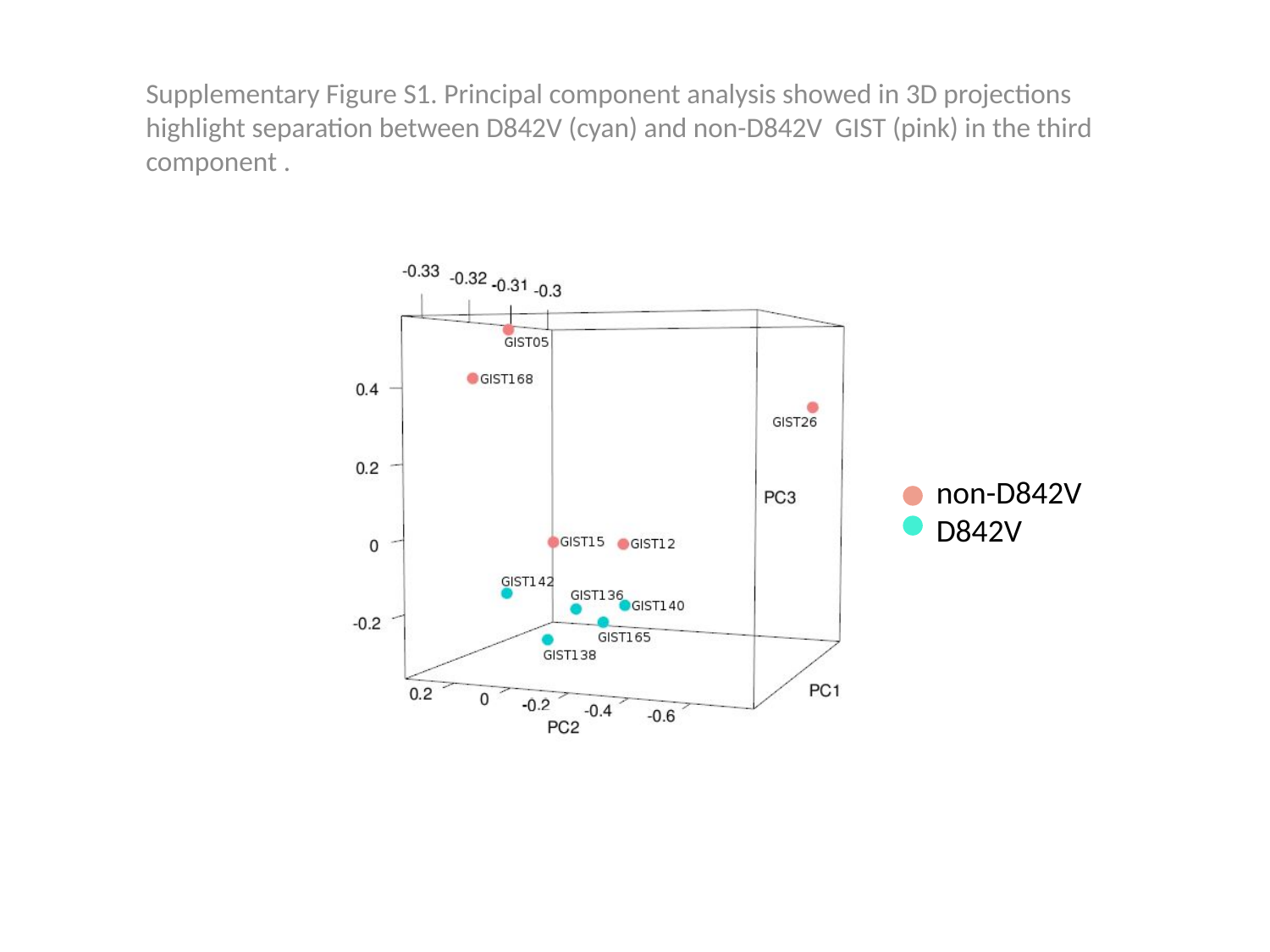

Supplementary Figure S1. Principal component analysis showed in 3D projections highlight separation between D842V (cyan) and non-D842V GIST (pink) in the third component .
non-D842V
D842V

## Slide 2
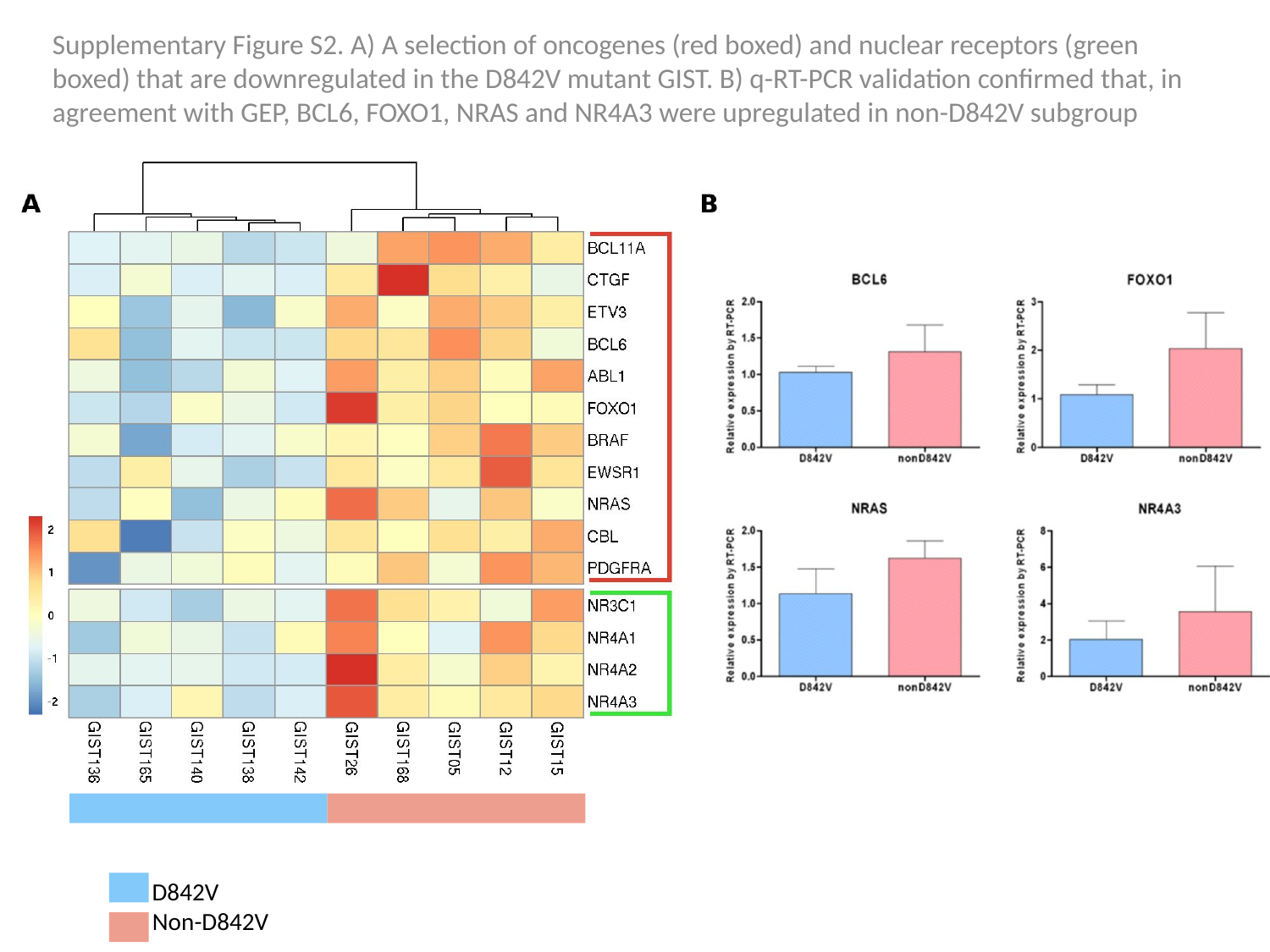

Supplementary Figure S2. A) A selection of oncogenes (red boxed) and nuclear receptors (green boxed) that are downregulated in the D842V mutant GIST. B) q-RT-PCR validation confirmed that, in agreement with GEP, BCL6, FOXO1, NRAS and NR4A3 were upregulated in non-D842V subgroup
D842V
Non-D842V

## Slide 3
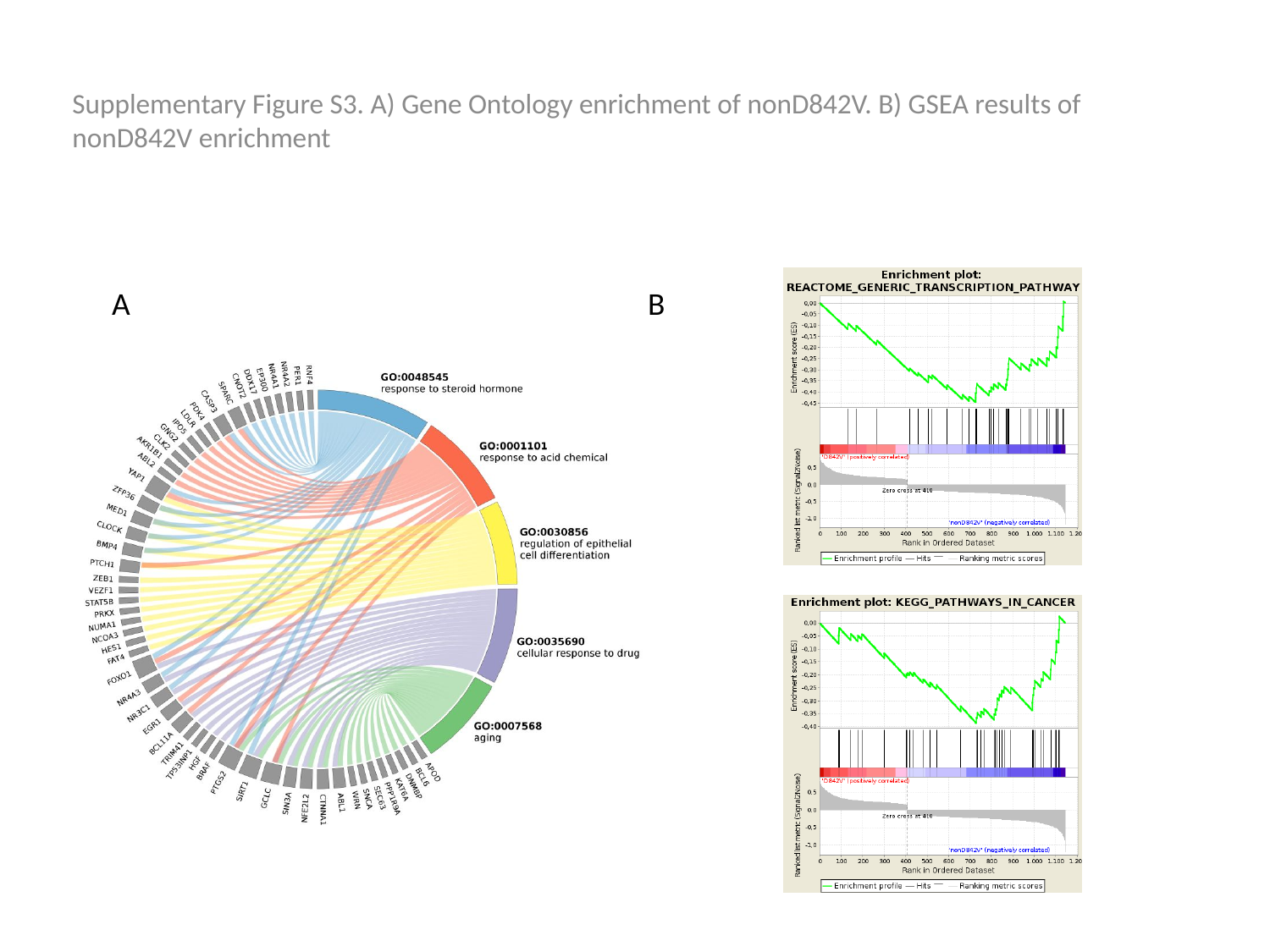

Supplementary Figure S3. A) Gene Ontology enrichment of nonD842V. B) GSEA results of nonD842V enrichment
A
B

## Slide 4
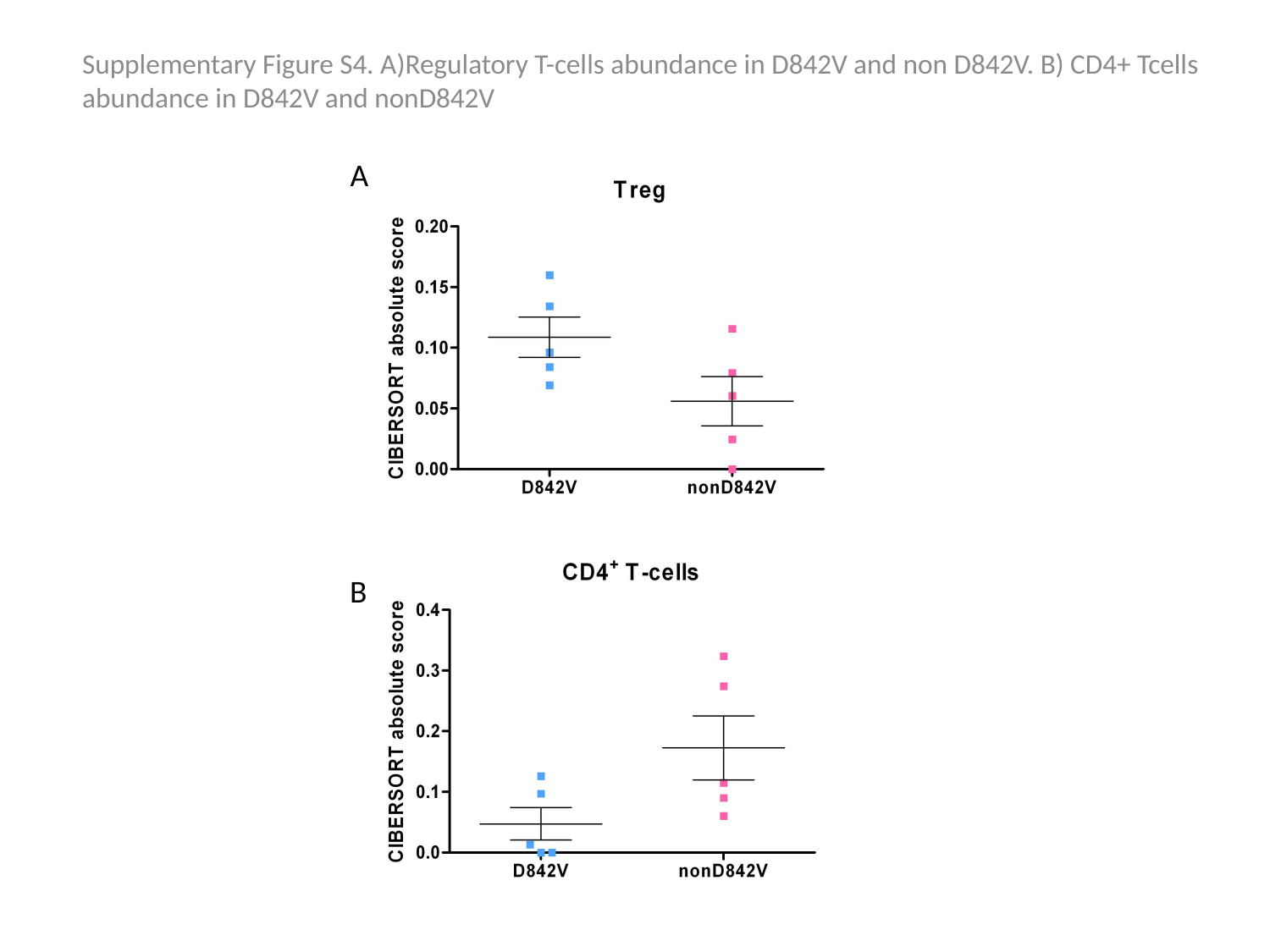

Supplementary Figure S4. A)Regulatory T-cells abundance in D842V and non D842V. B) CD4+ Tcells abundance in D842V and nonD842V
A
B

## Slide 5
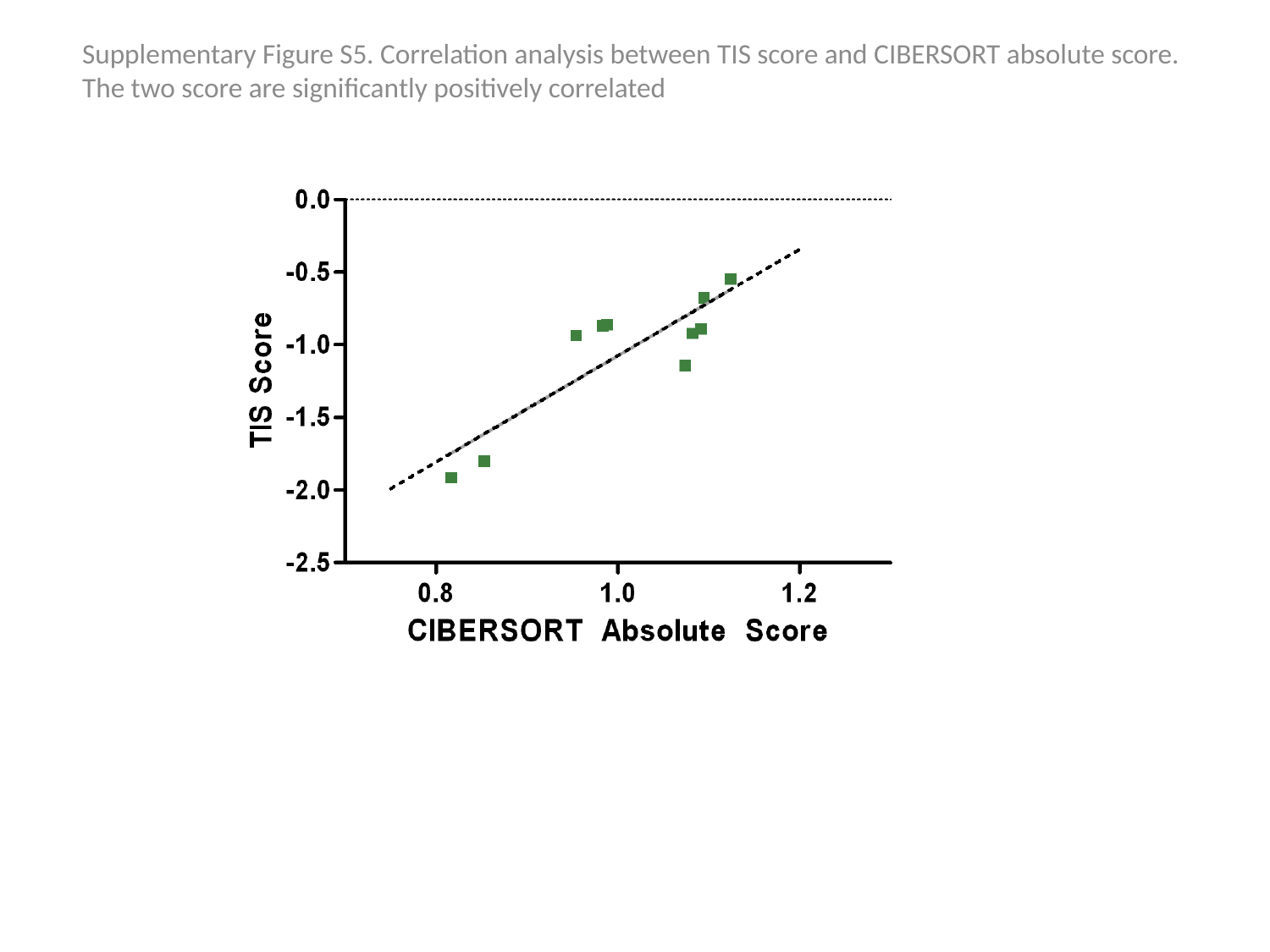

Supplementary Figure S5. Correlation analysis between TIS score and CIBERSORT absolute score. The two score are significantly positively correlated
